# Bi-frontal pneumocephalus is an independent risk factor for early postoperative agitation in adult patients admitted to intensive care unit after elective craniotomy for brain tumor: A prospective cohort study

**DOI:** 10.1371/journal.pone.0201064

**Published:** 2018-07-19

**Authors:** Hua-Wei Huang, Li-Mei Yan, Yan-Lin Yang, Xuan He, Xiu-Mei Sun, Yu-Mei Wang, Guo-Bin Zhang, Jian-Xin Zhou

**Affiliations:** 1 Department of Critical Care Medicine, Beijing Tiantan Hospital, Capital Medical University, Beijing, China; 2 Department of Critical Care Medicine, Inner Mongolia People’s Hospital, Hohhot, Inner Mongolia, China; 3 Department of Neurosurgery, Beijing Tiantan Hospital, Capital Medical University, Beijing, China; Jinling Clinical Medical College of Nanjing Medical University, CHINA

## Abstract

Postoperative agitation frequently occurs after general anesthesia and may be associated with serious consequences. However, studies in neurosurgical patients have been inadequate. We aimed to investigate the incidence and risk factors for early postoperative agitation in patients after craniotomy, specifically focusing on the association between postoperative pneumocephalus and agitation. Adult intensive care unit admitted patients after elective craniotomy under general anesthesia were consecutively enrolled. Patients were assessed using the Sedation-Agitation Scale during the first 24 hours after operation. The patients were divided into two groups based on their maximal Sedation-Agitation Scale: the agitation (Sedation-Agitation Scale ≥ 5) and non-agitation groups (Sedation-Agitation Scale ≤ 4). Preoperative baseline data, intraoperative and intensive care unit admission data were recorded and analyzed. Each patient’s computed tomography scan obtained within six hours after operation was retrospectively reviewed. Modified Rankin Scale and hospital length of stay after the surgery were also collected. Of the 400 enrolled patients, agitation occurred in 13.0% (95% confidential interval: 9.7–16.3%). Body mass index, total intravenous anesthesia, intraoperative fluid intake, intraoperative bleeding and transfusion, consciousness after operation, endotracheal intubation kept at intensive care unit admission and mechanical ventilation, hyperglycemia without a history of diabetes, self-reported pain and postoperative bi-frontal pneumocephalus were used to build a multivariable model. Bi-frontal pneumocephalus and delayed extubation after the operation were identified as independent risk factors for postoperative agitation. After adjustment for confounding, postoperative agitation was independently associated with worse neurologic outcome (odd ratio: 5.4, 95% confidential interval: 1.1–28.9, P = 0.048). Our results showed that early postoperative agitation was prevalent among post-craniotomy patients and was associated with adverse outcomes. Improvements in clinical strategies relevant to bi-frontal pneumocephalus should be considered.

**Trial registration:** ClinicalTrials.gov (NCT02318199).

## Introduction

Postoperative agitation after general anesthesia has been reported to occur in 3.7 to 29% of patients and may be associated with serious consequences, such as unplanned extubation, injuries and longer post-anesthesia care unit stay [1–7]. During the early stage of anesthesia recovery, neurosurgical patients may be more vulnerable to stress caused by agitation [8, 9]. Physiological fluctuations may result in edema, hemorrhage, and, ultimately, ischemia [10]. In our previous pilot study, which included 123 patients who underwent elective intracranial operations, we found the incidence of agitation within 12 hours postoperatively to be 29%, which was higher than that previously observed in other surgical populations [4]. However, investigations of this phenomenon in neurosurgical populations have been inadequate [1, 3, 5–7].

The most commonly reported independent risk factors for postoperative agitation in non-neurosurgical patients, including longer anesthesia duration, delayed extubation and pain, have also been found to present in neurosurgical patients [4, 8, 9]. However, brain lesions and intracranial manipulations in neurosurgical patients might affect the brain regions which involves cognition and emotion, and are assumed to influence postoperative cognition [4]. Moreover, during our clinical work, we noticed that post-craniotomy frontal pneumocephalus might also serve as a potential risk factor. Due to the preventable nature of pneumocephalus, further investigation is needed to clarify its relationship with agitation. Therefore, we conducted this prospective cohort study of adult patients who had undergone elective craniotomy for brain tumors. Incidence and clinical consequences of early postoperative agitation were documented. We aimed to investigate the risk factors for agitation, specifically focusing on the association between postoperative pneumocephalus and agitation. The association of agitation with long-term outcomes was also analyzed.

## Materials and methods

### Study design, ethics and patient population

This prospective cohort study was approved by the Institutional Review Board of Beijing Tiantan Hospital, Beijing, China (KY2014-034-01). Written informed consents were obtained from study subjects or their pre-defined healthcare decision makers. The study protocol was registered at ClinicalTrials.gov (NCT02318199; https://clinicaltrials.gov/ct2/show/NCT02318199). Three authors (HWH, LMY and JXZ) had access to information that could identify individual participants during and after data collection.

The study was conducted in a neurosurgical ICU of a University affiliated hospital (Beijing, China) between Jan 1 and Aug 31, 2015. Patients admitted to the ICU after intracranial operations under general anesthesia were consecutively screened for study eligibility. The inclusion criterion was adult patients who had undergone elective craniotomy for brain tumors. The exclusion criteria included: 1) aged under 18 or over 80 years; 2) preoperative impairment of consciousness; 3) not following simple commands (lift hands or open mouth) during the first 24 hours after the operation; and 4) interval longer than 24 hours between the end of the operation and ICU admission.

### Routine practices of neurosurgery, anesthesia and postoperative care

During the study, no attempts were made to change the standard of care, and routine practices were followed.

In our institute, all neurosurgeons had to undergo standard pre-job training for intracranial operation, which included the instruction of adequate air removal via physical saline injection prior to dural closure.

All intracranial surgeries were performed under general balanced anesthesia or total intravenous anesthesia (TIVA). Anesthesia was induced with intravenous propofol and sufentanil or remifentanil. Tracheal intubation was facilitated with intravenous rocuronium or cisatracurium. Anesthesia was maintained with propofol and/or sevoflurane or isoflurane. Sufentanil or remifentanil was administered intermittently or continuously, as needed. Muscle relaxant was administered according to train-of-four monitoring. The choice of agents was at the discretion of the anesthesiologist. For adult patients with brain tumors scheduled elective operation, the anesthesiologist and the neurosurgeon discussed postoperative ICU admission using criteria including, but were not limited to, age over 65 years, American Society of Anesthesiology (ASA) classification III or higher, large tumor or located at the brain stem, preoperative midline shift, preoperative consciousness impairment, anticipated prolonged operation and anticipated delayed extubation. At the end of the operation, the anesthesiologist and the neurosurgeon also discussed whether the patient had reasons for unplanned ICU admission, mainly including unanticipated delayed extubation, major intraoperative bleeding or brain swelling, injury to cranial nerve IX, X and XII, and severe cardiorespiratory instability during the attempt of emergence. Approximately half of the patients who had undergone elective craniotomy were admitted to the ICU for overnight postoperative monitoring.

In the ICU, nurses evaluated the patients’ Glasgow Coma Scale (GCS) scores and focal signs and performed pupillary examinations hourly or as needed. Most patients received patient-controlled intravenous analgesia (PCIA), which was composed of sufentanil 100 μg and tropisetron 10 mg in 100 ml 0.9% sodium chloride solution. A basal PCIA infusion (2 ml/h) is started after the confirmation of the patient’s cardiorespiratory stability and recovery of consciousness. A computed tomography (CT) scan was routinely performed within six hours postoperatively. All extubated patients received supplemental oxygen by simple face mask at flow of 3–6 L/min. In patients with delay extubation, oxygen was delivered by T tube. Mechanical ventilation was initiated when the patient could not maintain adequate spontaneous breathing or the pulse oxygenation saturation (SpO_2_) below 90%. Dexmedetomidine was continuously infused (0.4 μg/kg/h) in mechanically ventilated patients until they were weaned off the ventilator [10]. The presence of agitation and depth of sedation were evaluated using the Sedation-Agitation Scale (SAS) [11], which had been incorporated into clinical practice at our facility for more than three years [4]. All ICU physicians and nurses were trained to perform SAS assessment. Both nurses and physicians carefully evaluated patients with agitation (SAS score from 5 to 7) and excluded the possibility of organic causes of agitation, including pain, acute deterioration of cardiorespiratory function, a new neurologic event and hypoglycemia. When patients complained of pain, an intravenous bolus of fentanyl (25 μg) was administered. An intermittent or continuous intravenous infusion of midazolam was used for agitated patients, and the level of sedation was titrated until a SAS score of 3 to 4 was achieved. In patients with delayed extubation, after recovery from anesthesia, the ICU physician evaluated the patient’s reliability for extubation by a screening checklist, including assessments of consciousness, cardiorespiratory status, muscle strength recovery, gag reflex and cough function [12]. When each item in the checklist was passed, endotracheal extubation was performed by registered ICU nurses. Most patients were discharged from the ICU in the next morning after achieving normal neurological, hemodynamic and respiratory statuses.

### Agitation assessment and definition

During the study period, four trained investigators took turns screening and evaluating the SAS scores of the patients. The SAS score of each eligible patient was evaluated hourly until the patient was capable of following commands (SAS ≥ 3). After enrolment, SAS scores were assessed every four hours or as needed until either 24 hours of evaluation or ICU discharge, whichever occurred first. Agitation was defined as a SAS score ranging from 5 to 7 [11]. The patients were divided into two groups based on their maximal SAS score: the agitation group (maximal SAS ≥ 5) and the non-agitation group (maximal SAS ≤ 4).

### Assessment of postoperative CT scans

**Fig 1:**
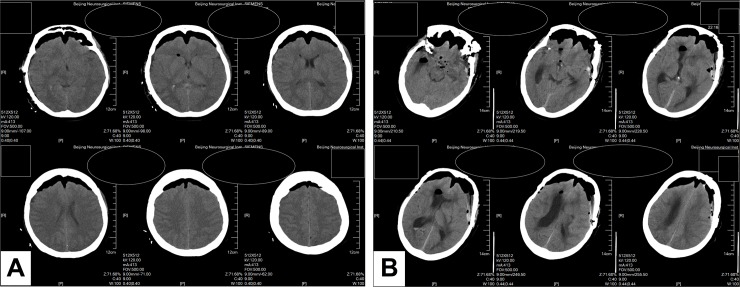
**Example CT scans identified as bi-frontal pneumocephalus (A) and “Mount Fuji sign” (B).** Patients name and medical record number are masked. Bi-frontal pneumocephalus (A) was defined as complying with all three: 1) subdural areas of hypoattenuation were bilaterally presented in frontal region; 2) low attenuation mass existed in at least four serial axial slices (1 cm thickness); and 3) the maximal displacement of the frontal lobe from the dura was more than 1 cm at least in one slice. “Mount Fuji sign” (B) was identified by bilateral subdural hypoattenuating collections, collapsed frontal lobes and widening of the interhemispheric space between the tips of the frontal lobes.

Two attending neuroradiologists who were blinded to agitation status retrospectively reviewed each patient’s first postoperative CT scans. Bi-frontal subdural pneumocephalus was defined by the following criteria: 1) subdural areas of hypoattenuation presenting bilaterally in the frontal region; 2) low attenuation mass presence in at least four serial axial slices (1 cm thickness); and 3) maximal displacement of the frontal lobe from the dura of more than 1 cm in at least one slice (**Fig 1A**) [13, 14]. A special type of bi-frontal pneumocephalus, the “Mount Fuji sign”, was characterized by bilateral subdural hypoattenuating collections, collapsed frontal lobes and widening of the interhemispheric space between the tips of the frontal lobes (**Fig 1B**) [15]. The “Mount Fuji sign” was deliberately inspected. Postoperative hematomas, ischemia and midline shifts were also observed. Two neuroradiologists independently reviewed the CT scans. Discrepancies were resolved by discussion until consensus was reached.

### Data collection

During the study period, the numbers of elective craniotomies performed for brain tumors and patients transferred to the ICU and neurosurgical ward were documented. The time of ICU admission and time interval between admission and enrolment were also recorded.

Baseline and preoperative data were extracted from hospital records, including the patients’ demographic characteristics, ASA classification, health history (comorbid diseases, smoking and alcohol abuse, and long-term use of antidepressant drugs or benzodiazepines). The type of tumor (glioma or non-glioma) was documented based on the postoperative pathological reports issued during the follow-up period.

Intraoperative data were extracted from anesthesia and operation records, including tumor location (supratentorial or infratentorial), surgical approach (frontal or non-frontal), anesthesia duration and method (balanced anesthesia or TIVA), fluid balance, bleeding amount, transfusion occurrence, mannitol and steroid use, and hypotension episode occurrence.

ICU admission data were extracted from nursing records, including GCS (especially scores on the motor response subscale), body temperature, endotracheal tube kept at ICU admission and need for mechanical ventilation, central venous catheterization, external ventricular drainage tube and PCIA device placement, SpO_2_ below 90% and serum glucose concentration above 10 mmol/L (especially in patients without a history of diabetes). Complaints of pain during screening period before the enrollment were also documented.

Patients were followed up until hospital discharge, death or 90 days after enrolment, whichever occurred first. Following data were collected from ICU nursing and hospital records, including self-extubation and accidental removal of catheter in the ICU, duration of mechanical ventilation, time of endotracheal extubation, occurrence of reintubation and tracheotomy, unexpected reoperation within 72 hours of surgery, and use of sedatives and fentanyl during ICU stay. Long-term outcomes, including hospital LOS after the surgery and modified Rankin Scale (mRS) scores, were collected at the end of follow-up. Poor neurologic outcome was defined as a mRS score of 5 or 6 [16]. Results of the retrospective analyses of the first postoperative CT scans were documented.

### Statistical analysis

Categorical variables are expressed as counts (percentages), and continuous data are reported as medians with interquartile ranges (IQRs). Missing data and loss to follow-up were documented. The incidence of post-operative agitation and 95% confidential interval (CI) were calculated. The agitation and non-agitation groups were univariably compared to screen for potential confounding variables in a multivariable model predicting agitation. The associations of preoperative baseline variables, intraoperative variables, ICU admission data and CT scan data with the agitation were assessed. Categorical variables were compared using two-tailed Pearson chi-square tests, and Fisher’s exact tests were performed for variables with small cell counts. Continuous variables were compared using the Mann-Whitney U test. The possible interaction between variables was analyzed. Factors with P values < 0.05 in the univariable analysis were entered into the multivariable analysis with stepwise backward logistic regression to identify independent risk factors for agitation. Odds ratios (ORs) and 95% CIs were used to assess the independent contributions of significant factors. The Hosmer-Lemeshow test was used to determine whether the model fitted the data adequately well. The association of agitation with hospital LOS after the surgery and poor neurologic outcome was analyzed using a multivariable logistic regression analysis by confounding adjustment.

Statistical analyses were performed using SPSS 20.0 (SPSS Inc., Chicago, IL, USA). A P value of less than 0.05 was considered statistically significant.

According to standard recommendations, 10 cases of interest (agitation) would be required for each degree of freedom in the multivariable model to reliably fit the model. Therefore, a model with 4 degrees of freedom would require at least 40 patients with postoperative agitation [4]. We anticipated an average incidence of agitation in the present cohort using data from previous reports (11%) [1–7], and planned to enroll 400 cases to identify risk factors for postoperative agitation.

## Results

**Fig 2:**
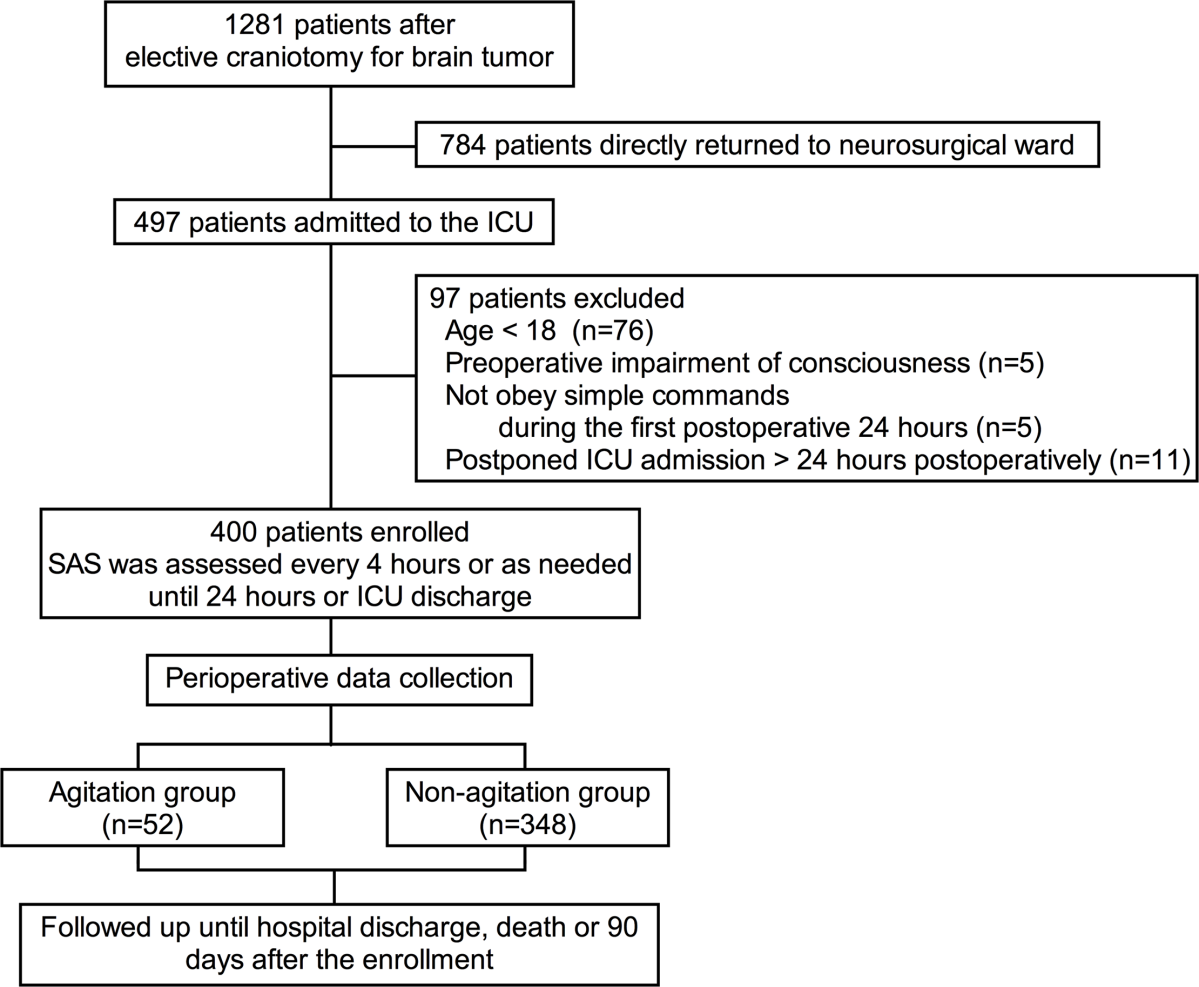
The patients flow diagram. Abbreviations: ICU, intensive care unit; SAS, Sedation-Agitation Scale.

During the study period, 1281 patients underwent elective craniotomy for brain tumors, of whom 784 returned to the neurosurgical ward and 497 were admitted to the ICU. After excluding 97 patients, 400 patients were included (**Fig 2**). All patients were enrolled within six hours of ICU admission, with 258/400 (64.5%) patients enrolled just after admission, 131/400 (32.8%) patients enrolled between one and three hours after admission and 11/400 (2.7%) patients enrolled between four and six hours after admission. Three hundred sixty-one patients (361/400, 90.3%) were discharged on postoperative day 1, and the remaining patients (39/400, 9.7%) stayed in the ICU for longer than 24 hours.

### Incidence of postoperative agitation

A total of 2019 SAS assessments were performed, with an average of five assessments per patient. SAS scores were ≥ 5 on 89/2019 (4.4%, 95% CI: 3.5–5.3%) of these assessments. According to the individual data, 300/400 (75.0%), 48/400 (12.0%), 21/400 (5.3%), 24/400 (6.0%) and 7/400 (1.8%) patients had a maximal SAS score of 3, 4, 5, 6 and 7, respectively. Thus, at least one episode of agitation (SAS ≥ 5) occurred in 52/400 (13.0%, 95% CI: 9.7–16.3%) patients.

### Risk factors for postoperative agitation

In the univariable analyses, compared with the non-agitation group, patients in the agitation group had significantly higher body height and weight, intraoperative fluid intake, and amount of bleeding (and hourly bleeding); a significantly lower GCS score at ICU admission; and significantly more use of balanced anesthesia and higher occurrence of transfusion, endotracheal tube kept at ICU admission, need for mechanical ventilation, serum glucose concentration ≥ 10 mmol/L in patients without a history of diabetes, complaint of pain during screening, and bi-frontal pneumocephalus (**Tables 1–3**). The “Mount Fuji sign” was identified in four cases (4/400, 1.0%), all in the non-agitation group and responsive to conservative therapy without operation. Bi-frontal pneumocephalus occurred more frequently in patients who underwent frontal approach than non-frontal approach operations (57/159 [35.8%] versus 33/241 [13.7%], P < 0.001), and in patients who underwent surgery for supratentorial tumors than for infratentorial tumors (58/209 [27.8%] versus 32/191 [16.8%], P = 0.009).

**Table 1:** Univariable analyses of baseline and preoperative data.

Variables	All patients (n = 400)	Agitation (n = 52)	Non-agitation (n = 348)	P
Male sex (n [%])	169 (42.3)	26 (50.0)	143 (41.1)	0.225
Age (years; Median [IQR])	48 (38–56)	51 (36–56)	47 (38–56)	0.652
Height (cm; Median [IQR])	165 (160–171)	168 (160–175)	165 (160–170)	0.023
Weight (kg; Median [IQR])	66 (60–75)	75 (61–83)	65 (59–75)	0.007
BMI (kg/m^2^; Median [IQR])	24.4 (22.1–26.7)	25.7 (22.4–27.3)	24.2 (22.0–26.6)	0.064
ASA classifications (n [%])				0.174
I	32 (8.0)	5 (9.6)	27 (7.8)	
II	347 (86.8)	43 (82.7)	304 (87.4)	
III	20 (5.0)	3 (5.8)	17 (4.9)	
IV	1 (0.3)	1 (1.9)	0 (0.0)	
History of smoking (n [%])	41 (10.3)	9 (17.3)	32 (9.2)	0.085
History of alcohol abuse (n [%])	12 (3.0)	2 (3.8)	10 (2.9)	0.660
History of ADD and/or benzodiazepines (n [%])	2 (0.5)	1 (1.9)	1 (0.3)	0.243
Type of tumor (n [%])				0.475
Gliomas	43 (10.8)	7 (13.5)	36 (10.3)	
Non-gliomas	357 (89.3)	45 (86.5)	312 (89.7)	
Medical history (n [%])	108 (27.0%)	16 (30.8)	92 (26.4)	0.512
History of hypertension	87 (21.8)	15 (28.8)	72 (20.7)	0.207
History of CAD	2 (0.5)	0 (0)	2 (0.6)	> 0.999
History of cardiac arrhythmia	7 (1.8)	0 (0)	7 (2.0)	0.602
History of ischemic stroke	5 (1.3)	0 (0)	5 (1.4)	> 0.999
History of diabetes mellitus	25 (6.3)	3 (5.8)	22 (6.3)	> 0.999

Abbreviations: ADD, antidepressant drugs; ASA, American Society of Anesthesiologist; BMI, body mass index; CAD, coronary artery disease; ICU, intensive care unit; IQR, interquartile range.

**Table 2:** Univariable analyses of intraoperative data.

Variables	All patients (n = 400)	Agitation (n = 52)	Non-agitation (n = 348)	P
Frontal approach (n [%])	159 (39.8)	22 (42.3)	137 (39.4)	0.686
Location of the tumor (n [%])				0.960
Supratentorial	209 (52.3)	27 (51.9)	182 (52.3)	
Infratentorial	191 (47.6)	25 (48.1)	166 (47.7)	
Duration of anesthesia (hours; Median [IQR])	5.7 (4.7–6.8)	6.0 (5.0–7.3)	5.7 (4.7–6.7)	0.085
Method of anesthesia (n [%])				0.034
TIVA	93 (23.3)	6 (11.5)	87 (25.0)	
Balanced anesthesia	307 (76.7)	46 (88.5)	261 (75.0)	
Use of opioids for anesthesia maintain (n [%])				0.879
Only remifentanil	326 (81.5)	43 (82.7)	283 (81.3)	
Only sufentanil	28 (7.0)	4 (7.7)	24 (6.9)	
Both remifentanil and sufentanil	46 (11.5)	5 (9.6)	41 (11.8)	
Fluid balance (ml; Median [IQR])	1500 (11001988)	1650 (1125–2280)	1500 (1100–1925)	0.258
Fluid intake (ml; Median [IQR])	3500 (2850–4000)	3500 (3005–4750)	3425 (2750–4000)	0.049
Fluid intake per hour (ml; Median [IQR])	600 (509–714)	604 (529–747)	599 (500–711)	0.506
Fluid output (ml; Median [IQR])	1800 (1300–2700)	2000 (1300–2900)	1775 (1300–2600)	0.222
Fluid output per hour (ml; Median [IQR])	324 (240–434)	333 (250–421)	320 (239–435)	0.627
Amount of bleeding (ml; Median [IQR])	300 (200–500)	500 (200–1000)	300 (200–500)	0.005
Amount of bleeding per hour (ml; Median [IQR])	71 (48–113)	80 (57–163)	70 (48–103)	0.018
Bleed transfusion (n [%])	117 (29.3%)	23 (44.2%)	94 (27.0%)	0.011
Use of mannitol (n [%])	147 (36.8%)	19 (36.5%)	128 (36.8%)	0.973
Use of steroid (n [%])	36 (9.0%)	4 (7.7%)	32 (9.2%)	> 0.999
Episode of hypotension (n [%])	137 (34.3%)	16 (30.8%)	121 (34.8%)	0.571

Abbreviations: IQR, interquartile range; TIVA, total intravenous anesthesia.

**Table 3:** Univariable analyses of ICU admission data.

Variables	All patients (n = 400)	Agitation (n = 52)	Non-agitation (n = 348)	P
GCS at ICU admission (Median [IQR])	14 (3–14)	6 (3–14)	14 (4–14)	< 0.001
Motor responses in GCS (Median [IQR])	6 (1–6)	4 (1–6)	6 (2–6)	< 0.001
Body temperature at ICU admission °C (n [%])	36.3 (36.0–37.0)	36.4 (36.0–37.3)	36.3 (36.0–37.0)	0.259
Below 36°C (n [%])	69 (17.3)	6 (11.5)	63 (18.1)	0.325
Endotracheal tube kept (n [%])	102 (25.5)	28 (53.8)	74 (21.3)	< 0.001
Need for mechanical ventilation (n [%])	14 (3.5)	5 (9.6)	9 (2.6)	0.025
CVC (n [%])	323 (80.8)	44 (84.6)	279 (80.2)	0.448
EVD tube (n [%])	9 (2.3)	2 (3.8)	7 (2.0)	0.331
PCIA (n [%])	330 (82.5)	38 (73.1)	292 (83.9)	0.055
SpO_2_ < 90% (n [%])	9 (2.3)	0 (0)	9 (2.6)	0.612
Serum glucose ≥10mmol/L (n [%])	59 (14.8%)	12 (23.1)	47 (13.5)	0.069
Without diabetes history (n [%])	47 (11.8%)	11 (21.2)	36 (10.3)	0.024
Complaint of pain (n [%])	35 (8.8%)	9 (17.3)	26 (7.5)	0.031
First postoperative CT scan (n [%])				
Hematoma	18 (4.5)	2 (3.8)	16 (4.6)	>0.999
Ischemia	12 (3.0)	2 (3.8)	10 (2.9)	0.661
Midline shift	26 (6.5)	6 (11.5)	20 (5.8)	0.129
Bi-frontal pneumocephalus	90 (22.5)	22 (42.3)	68 (19.5)	<0.001

Abbreviations: CT, computed tomography; CVC, central venous catheter; EVD, external ventricular drainage; GCS, Glasgow Coma Scale; ICU, intensive care unit; IQR, interquartile range; PCIA, patient-controlled intravenous analgesia; SpO_2_, pulse oxygenation saturation.

In the multivariable analyses, we combined body height and weight as body mass index (**Table 1**, P = 0.064). We included the hourly amount of bleeding variable (**Table 2**, P = 0.018) to avoid the influence of the anesthesia duration variable. To clarify the interaction between bleeding and transfusion, we arbitrarily stratified patients into two clusters according to the median of the hourly bleeding variable (71 ml/h) as having major (> 71 ml/h) and minor intraoperative bleeding (≤ 71 ml/h). We then divided the patients into three classifications: 1) minor bleeding; 2) major bleeding without transfusion; and 3) major bleeding with transfusion (**Table 4**). By combining the endotracheal tube and mechanical ventilation variables, we categorized patients into the following groups: 1) extubated in the operating room; 2) endotracheal tube kept at ICU admission without the need for mechanical ventilation; and 3) endotracheal tube kept at ICU admission with the need for mechanical ventilation (**Table 4**). The classification of the intraoperative bleeding and transfusion as well as endotracheal tube and mechanical ventilation variables were included as a categorical covariate in the multivariable models.

**Table 4:** Covariate classifications used for in the multivariate model building.

Classifications	All patients (n = 400)	Agitation (n = 52)	Non-agitation (n = 348)	P
Hourly bleeding and transfusion				0.008
Minor bleeding	197 (49.3)	22 (42.3)	175 (50.3)	
Major bleeding without transfusion	97 (23.5)	7 (13.5)	90 (25.9)	
Major bleeding with transfusion	106 (26.5)	23 (44.2)	83 (23.8)	
Delayed extubation and mechanical ventilation				<0.001
Extubated at operating room	298 (74.5)	24 (46.2)	274 (78.7)	
Endotracheal kept at ICU admission without the need of MV	88 (22.0)	23 (44.2)	65 (18.7)	
Endotracheal kept at ICU admission with the need of MV	14 (3.5)	5 (9.6)	9 (2.6)	

Data are shown as n (%).

Abbreviations: ICU, intensive care unit; MV, mechanical ventilation.

The results of the multivariable analysis showed that bi-frontal pneumocephalus and endotracheal tube kept at ICU admission (either without or with the need for mechanical ventilation) were independent risk factors for postoperative agitation (**Table 5**). The results of the Hosmer-Lemeshow test showed the model fits the data adequately well (P = 0.782).

**Table 5:** Independent risk factors for postoperative agitation.

Risk factors	Odd ratio (95% confidential interval)	P
Bi-frontal pneumocephalus	4.4 (2.2–8.7)	< 0.001
Intubation status and mechanical ventilation		0.001
Extubated at operating room	1 (reference)	
Endotracheal tube kept at ICU admission without the need of MV	3.6 (1.8–7.4)	< 0.001
Endotracheal tube kept at ICU admission with the need of MV	5.2 (1.4–19.0)	0.014

The Hosmer-Lemeshow test: P = 0.782.

Abbreviations: ICU, intensive care unit; MV, mechanical ventilation.

### Outcomes

Patients were followed up at a median (IQR) of 10 (8–14) days after the operation. Follow-up data are shown in **Table 6**. Multivariable logistic regression analysis revealed that after adjusting for significant potential confounders, including GCS at ICU admission, endotracheal tube kept at ICU admission, and serum glucose concentration ≥ 10 mmol/L in patients without a history of diabetes, postoperative agitation was independently associated with poor-grade mRS (OR: 5.4, 95% CI: 1.1–28.9, P = 0.048) (**Table 7**). However, after adjusting for significant potential confounders, including GCS at ICU admission, motor function of GCS at ICU admission and endotracheal tube kept at ICU admission, postoperative agitation was not independently associated with longer hospital length of stay (LOS > 10 days) (OR: 1.4, 95% CI: 0.7–2.8, P = 0.294) (**Table 8**).

**Table 6:** Follow-up data.

Variables	All patients (n = 400)	Agitation (n = 52)	Non-agitation (n = 348)
Accident removal of ET and CVC (n [%])	3 (0.8)	3 (5.8)	0 (0)
Duration of MV (hours; median [IQR])	11 (5–24) (n = 14)	12 (8–24) (n = 5)	9 (5–24) (n = 9)
Duration of intubation (hours; median [IQR])	16 (16–39) (n = 102)	16 (14–22) (n = 28)	16 (16–48) (n = 74)
Tracheostomy (n [%])	15 (14.7%) (n = 102)	4 (14.3%) (n = 28)	11 (14.9%) (n = 74)
Re-intubation (n [%])	3 (2.9%) (n = 102)	1 (3.6%) (n = 28)	2 (2.7%) (n = 74)
Use of sedatives (n [%])	40 (10.0)	33 (63.5)	7 (2.0)
Only dexmedetomidine (n [%])	15 (3.8)	9 (17.3)	6 (1.7)
Only midazolam (n [%])	23 (5.8)	22 (42.3)	1 (0.3)
Combined dexmedetomidine and midazolam (n [%])	2 (0.5)	2 (3.8)	0 (0)
Use of fentanyl (n [%])	28 (7.0)	9 (17.3)	19 (5.5)
ICU discharge at postoperative day 1 (n [%])	361 (90.3)	44 (84.6)	317 (91.1)
Unexpected re-operation within 72 hours (n [%])	8 (2.0%)	1 (1.9%)	7 (2.0%)

Abbreviations: CVC, central venous catheter; ET, endotracheal tube; ICU, intensive care unit; IQR, interquartile range; MV, mechanical ventilation.

**Table 7:** Risk factors for unfavorable functional neurologic outcome (adjusted analysis).

Variables	Adjusted odd ratio (95% CI)	P
Agitation	5.4 (1.1–28.9)	0.048
Serum glucose ≥ 10mmol/L without diabetes history	6.2 (1.2–33.1)	0.033
GCS at ICU admission	1.9 (0.7–5.3)	0.231
Endotracheal tube kept at ICU admission	0.8 (0.1–5.9)	0.858

Abbreviations: CI, confidential interval; ICU, intensive care unit; GCS, Glasgow Coma Scale.

**Table 8:** Risk factors for longer hospital length of stay (adjusted analysis).

Variables	Adjusted odd ratio (95% CI)	P
Agitation	1.4 (0.7–2.8)	0.294
GCS at ICU admission	3.0 (1.6–5.9)	0.001
Motor response in GCS at ICU admission	1.3 (1.0–1.7)	0.056
Endotracheal tube kept at ICU admission	1.5 (0.8–2.9)	0.189

Abbreviations: CI, confidential interval; ICU, intensive care unit; GCS, Glasgow Coma Scale.

## Discussion

Our main findings were: 1) early postoperative agitation was prevalent in adult patients who had undergone elective craniotomy for brain tumors and was associated with adverse neurological outcomes; and 2) independent risk factors for agitation included bi-frontal pneumocephalus and delayed extubation, especially with the need for mechanical ventilation.

The incidence of agitation in our group of patients (13%) was within the range reported in previous studies (3% to 22%) [1, 3, 5–7, 17–20] but markedly lower than that identified in our previous study (29%) [4]. Several changes to clinical practice in our institution were implemented after the completion of our previous studies. We have incorporated SAS assessment into our daily clinical practice for more than three years [4]. Additionally, according to our previous investigation showing that prophylactic use of dexmedetomidine decreased the incidence of agitation in patients with delayed extubation [10], we routinely initiated continuous dexmedetomidine infusion at low dosage (0.4 μg/kg/h) in mechanically ventilated patients. However, whether these changes in our clinical strategies decreased the rate of agitation requires further investigation.

Previous studies found that postoperative delirium, including agitation, was associated with adverse outcomes [21]. We found that postoperative agitation was association with poor-grade mRS even after controlling the potential confounders. The relatively high prevalence of postoperative agitation and its association with adverse neurological outcome indicate the importance of clinical attention to agitated patients who have undergone elective craniotomy.

A number of risk factors for postoperative agitation have been reported in non-neurosurgical patients, including premedication, type of anesthesia, delayed extubation and pain [1, 3, 5–7]. In the present study, we found that delayed extubation, especially when occurring in combination with a need for mechanical ventilation, was associated with significantly increased risk of agitation. Although fast track anesthesia has been increasingly used in neurosurgical patients, delayed emergence is still advocated in patients with a high risk of postoperative complications [8, 9, 22, 23]. In our cohort, the prevalence of delayed extubation was 25.5% in patients who had been admitted to the ICU after elective craniotomy, and 40.8% in patients who had undergone infratentorial craniotomy. These data were similar to the results of previous studies [24–29]. Therefore, infratentorial tumors and delayed extubation were the major indications for postoperative ICU management, and clinical staff should be vigilant for the occurrence of agitation in these patients.

Pneumocephalus has been found to commonly occur during the early stage after craniotomy, but tension pneumocephalus requiring urgent surgical evacuation occurs rarely [30–34]. In our group of patients, the “Mount Fuji sign” only occurred in 1.0% of study subjects and no emergent operations were performed for intracranial air evacuation, a finding that was in accordance with previous case reports [32]. Appropriate closure of the dura is important for preventing pneumocephalus after craniotomy. The incidence of bi-frontal pneumocephalus (22.5%) in our cohort was lower than previously reported incidence of moderate-to-large pneumocephalus after craniotomy (approximately 50% to 70%) [30, 31]. In our institute, all neurosurgeons received standard pre-job training for intracranial operation, which included the standard procedure for dural closure. However, our results showed that bi-frontal pneumocephalus occurred more frequently in patients undergoing frontal approach (35.8%) and surgery for supratentorial tumor (27.8%), which indeed indicated the margin for further repeatedly reminding and training in dural closure technique.

Several case reports have suggested that agitation may occur in some patients with tension pneumocephalus [35, 36]. As expected, bi-frontal pneumocephalus was found to be an independent risk factor for postoperative agitation in our cohort of patients. In the supine position, gas in the subdural space tends to gather in the frontal region. Because the executive function of the frontal lobes involves cognition and emotion [37], stimulating the frontal lobes with a large volume of gas may result in abrupt changes in behavior, including agitation. However, this is only a presumptive mechanism and requires further investigation.

Given the preventable nature of large pneumocephalus after elective craniotomy and its relationship with postoperative agitation, several strategies might be adopted into routine clinical practice: 1) neurosurgeons should remain aware of the intricate processes involved with closing the skull, including adequate air removal via physical saline injection before dural closure and prior to carefully suturing the dura; 2) anesthesiologists should use nitrous oxide cautiously, especially during reoperation; and 3) ICU physicians should differentiate between agitation due to pneumocephalus and signs of elevated intracranial pressure. In addition to the use of sedatives and analgesics to control agitation, neurological status should be carefully monitored during the management of agitated patients with pneumocephalus to rule out or confirm the occurrence of tension pneumocephalus.

Previous evidence demonstrated that severe intraoperative hyperglycemia was a predictor of higher risk for postoperative infections in patients undergoing craniotomy [38]. In the present study, we determined serum glucose at the ICU admission, which may partly reflect the intraoperative hyperglycemia. We found that hyperglycemia during the early postoperative period, especially in patients without a history of diabetes, occurred more in agitated patients. Our data further enforced the alertness toward hyperglycemia in this population.There are limitations to the present study. First, we only enrolled ICU admitted patients. When compared with those directly returning to the neurosurgical ward, these patients might have had relatively higher ASA classifications, tumors that were more frequently infratentorially located, longer durations of anesthesia and greater amounts of bleeding during surgery. Thus, our patients represented a population that was at high-risk of postoperative agitation, and our results may limit the generalization to the entire population of patients undergoing craniotomy for brain tumors. Second, although we focused on the association between bi-frontal pneumocephalus and agitation, surgical factors potentially associated with pneumocephalus were not collected, such as the opening of the basal cistern and closing of the dura mater. The influence of these factors warrants further investigation. Third, we did not evaluate preoperatively neurological disorders and visual or hearing impairments because it could not be determined in advance which patients would be admitted to the ICU after surgery. Postoperative internal environmental disturbances, such as sleep disorders due to light and noise in the ICU, were not excluded. These factors may also be associated with postoperative agitation in the ICU [39]. Therefore, we cannot rule out a potential bias introduced by these missing data. Fourth, in the present study, we only evaluated postoperative agitation because there is no uniformly accepted tool for assessing delirium in brain injured patients. However, the Confusion Assessment Method (CAM) is increasingly employed in the neurological and neurosurgical patients [40]. Additionally, previous reports have suggested that hypoactive delirium might be more prevalent in the early period following general anesthesia [2, 6]. In the present study, the observation that patients with “Mount Fuji sign” type of pneumocephalus were not agitated might represent the occurrence of hypoactive delirium in this subgroup of patients. Therefore, we are ongoing a study focusing on the postoperative delirium in adult patients after elective intracranial operations to clarify the incidence and risk factors of delirium in this patient population (NCT03087838).

In conclusion, we found that early postoperative agitation was frequently identified in patients who had undergone elective craniotomy for brain tumors and was associated with adverse long-term neurological outcome. Risk factors for postoperative agitation included bi-frontal pneumocephalus and delayed extubation and mechanical ventilation. Improvements in the clinical strategies relevant to these risk factors should be considered, and further studies are needed to investigate the effect of such improvements on the occurrence of agitation and clinical outcomes.

## Supporting information

S1 File STROBE checklist. (PDF)
