# Supplementary material for: Bi-frontal pneumocephalus is an independent risk factor for early postoperative agitation in adult patients admitted to intensive care unit after elective craniotomy for brain tumor: A prospective cohort study
Source: PLoS One. 2018 Jul 19;13(7):e0201064. doi: 10.1371/journal.pone.0201064 (PMC6053234; doi:10.1371/journal.pone.0201064)
Supplement: S1 File — (PDF) [file pone.0201064.s001.pdf]

STROBE Checklist

	Item No.	Recommendation	Page No.	Relevant text from manuscript
**Title and abstract**	1	(*a*) Indicate the study's design with a commonly used term in the title or the abstract	1	Bi-frontal pneumocephalus is an independent risk factor for early postoperative agitation in adult patients after elective craniotomy for brain tumor: A prospective cohort study
		(*b*) Provide in the abstract an informative and balanced summary of what was done and what was found	2-3	Adult intensive care unit admitted patients after elective craniotomy under general anesthesia were consecutively enrolled……After adjustment for confounding, agitation was independently associated with worse neurologic outcome (odd ratio: 5.4, 95% confidential interval: 1.1–28.9, P = 0.048).
**Introduction**				
Background/rationale	2	Explain the scientific background and rationale for the investigation being reported	4	Postoperative agitation after general anesthesia has been reported to occur in 3.7 to 29 % of patients……Therefore, we conducted this prospective cohort study of adult patients who had undergone elective craniotomy for brain tumors.
Objectives	3	State specific objectives, including any prespecified hypotheses	5	We aimed to investigate the risk factors for agitation, specifically focusing on the association between postoperative pneumocephalus and agitation. The association of agitation with long-term outcomes was also analyzed.
**Methods**				
Study design	4	Present key elements of study design early in the paper	6	Study design, ethics and patient population<br>This prospective cohort study was approved by the Institutional Review Board of Beijing Tiantan Hospital, Beijing, China (KY2014-034-01).
Setting	5	Describe the setting, locations, and relevant dates, including periods of recruitment, exposure, follow-up, and data collection	6	The study was conducted in a neurosurgical ICU of University affiliated hospital (Beijing, China) between Jan 1 and Aug 31, 2015.
Participants	6	(*a*) *Cohort study*—Give the eligibility criteria, and the sources and methods of selection of participants. Describe methods of follow-up	6	The inclusion criterion was adult patients who had undergone elective craniotomy for brain tumors. The exclusion criteria included:……

		Case-control study—Give the eligibility criteria, and the sources and methods of case ascertainment and control selection. Give the rationale for the choice of cases and controls<br>*Cross-sectional study*—Give the eligibility criteria, and the sources and methods of selection of participants		
		(*b*) *Cohort study*—For matched studies, give matching criteria and number of exposed and unexposed<br>*Case-control study*—For matched studies, give matching criteria and the number of controls per case	9	The patients were divided into two groups based on their maximal SAS score: the agitation group (maximal SAS ≥ 5) and the non-agitation group (maximal SAS ≤ 4).
Variables	7	Clearly define all outcomes, exposures, predictors, potential confounders, and effect modifiers. Give diagnostic criteria, if applicable	9-12	Agitation assessment and definition<br>Assessment of postoperative CT scans<br>Data collection
Data sources/ measurement	8*	For each variable of interest, give sources of data and details of methods of assessment (measurement). Describe comparability of assessment methods if there is more than one group	9-12	The patients were divided into two groups based on their maximal SAS score: the agitation group (maximal SAS ≥ 5) and the non-agitation group (maximal SAS ≤ 4).
Bias	9	Describe any efforts to address potential sources of bias	9-12	Two attending neuroradiologists who were blinded to agitation status retrospectively reviewed each patient's first postoperative CT scans……Two neuroradiologists independently reviewed the CT scans. Discrepancies were resolved by discussion until consensus was reached.
Study size	10	Explain how the study size was arrived at		According to standard recommendations, 10 cases of interest (agitation) would be required……We anticipated an average incidence of agitation in the present cohort using data from previous reports (11%), and planned to enroll 400 cases to identify risk factors for postoperative agitation.

Continued on next page

Quantitative variables	11	Explain how quantitative variables were handled in the analyses. If applicable, describe which groupings were chosen and why	12	Categorical variables are expressed as counts (percentages), and continuous data are reported as medians with interquartile ranges (IQRs).
Statistical methods	12	(*a*) Describe all statistical methods, including those used to control for confounding	12-13	Statistical analysis
		(*b*) Describe any methods used to examine subgroups and interactions	12	The possible interaction between variables was analyzed.
		(*c*) Explain how missing data were addressed	12	Missing data and loss to follow-up were documented.
		(*d*) *Cohort study*—If applicable, explain how loss to follow-up was addressed<br>*Case-control study*—If applicable, explain how matching of cases and controls was addressed<br>*Cross-sectional study*—If applicable, describe analytical methods taking account of sampling strategy	12	Missing data and loss to follow-up were documented.
		(*e*) Describe any sensitivity analyses	12	The Hosmer-Lemeshow test was used to determine whether the model fitted the data adequately well.
Results				
Participants	13*	(a) Report numbers of individuals at each stage of study—eg numbers potentially eligible, examined for eligibility, confirmed eligible, included in the study, completing follow-up, and analysed	14	During the study period, 1281 patients……and Figure 2
		(b) Give reasons for non-participation at each stage	14	After excluding 97 patients, 400 patients were included.
		(c) Consider use of a flow diagram	14	Figure 2
Descriptive data	14*	(a) Give characteristics of study participants (eg demographic, clinical, social) and information on exposures and potential confounders	14-21	Table 1-3
		(b) Indicate number of participants with missing data for each variable of interest	14-21	Table 1-3
		(c) *Cohort study*—Summarise follow-up time (eg, average and total amount)	26	Follow-up data are shown in Table 6
Outcome data	15*	*Cohort study*—Report numbers of outcome events or summary measures over time	26	Table 6-8
		Case-control study—Report numbers in each exposure category, or summary measures of exposure		
		Cross-sectional study—Report numbers of outcome events or summary measures		
Main results	16	(*a*) Give unadjusted estimates and, if applicable, confounder-adjusted estimates and their precision (eg, 95% confidence interval). Make clear which confounders were adjusted for and why they were included	26	Table 7-8
		(*b*) Report category boundaries when continuous variables were categorized	26	Table 7-8
		(*c*) If relevant, consider translating estimates of relative risk into absolute risk for a meaningful time period	NA	

Continued on next page

Other analyses	17	Report other analyses done—eg analyses of subgroups and interactions, and sensitivity analyses	23	Table 4
**Discussion**				
Key results	18	Summarise key results with reference to study objectives	30	Our main findings were:……
Limitations	19	Discuss limitations of the study, taking into account sources of potential bias or imprecision. Discuss both direction and magnitude of any potential bias	33-34	There are limitations to the present study……
Interpretation	20	Give a cautious overall interpretation of results considering objectives, limitations, multiplicity of analyses, results from similar studies, and other relevant evidence	33	Thus, our patients represented a population that was at high-risk of postoperative agitation, and our results may limit the generalization to the entire population of patients undergoing craniotomy for brain tumors.
Generalisability	21	Discuss the generalisability (external validity) of the study results	33	Thus, our patients represented a population that was at high-risk of postoperative agitation, and our results may limit the generalization to the entire population of patients undergoing craniotomy for brain tumors.
**Other information**				
Funding	22	Give the source of funding and the role of the funders for the present study and, if applicable, for the original study on which the present article is based	In the submission window	

*Give information separately for cases and controls in case-control studies and, if applicable, for exposed and unexposed groups in cohort and cross-sectional studies.

Note: An Explanation and Elaboration article discusses each checklist item and gives methodological background and published examples of transparent reporting. The STROBE checklist is best used in conjunction with this article (freely available on the Web sites of PLoS Medicine at http://www.plosmedicine.org/, Annals of Internal Medicine at http://www.annals.org/, and Epidemiology at http://www.epidem.com/). Information on the STROBE Initiative is available at www.strobe-statement.org.
